# Role of Cofilin in Alzheimer’s Disease

**DOI:** 10.3389/fcell.2020.584898

**Published:** 2020-11-26

**Authors:** Qiang Wang, Wei Yuan, Xiaohang Yang, Yuan Wang, Yongfeng Li, Haifa Qiao

**Affiliations:** ^1^College of Acupuncture and Massage, Shaanxi University of Chinese Medicine, Xianyang, China; ^2^Shaanxi Key Laboratory of Acupuncture and Medicine, Xianyang, China; ^3^College of Medical Technology, Shaanxi University of Chinese Medicine, Xi’an, China; ^4^Xianyang Key Laboratory of Neurobiology and Acupuncture, Xi’an, China

**Keywords:** Alzheimer’s disease, Aβ, structure, function, regulation of cofilin

## Abstract

Alzheimer’s disease (AD) is a degenerative neurological disease and has an inconspicuous onset and progressive development. Clinically, it is characterized by severe dementia manifestations, including memory impairment, aphasia, apraxia, loss of recognition, impairment of visual-spatial skills, executive dysfunction, and changes in personality and behavior. Its etiology is unknown to date. However, several cellular biological signatures of AD have been identified such as synaptic dysfunction, β-amyloid plaques, hyperphosphorylated tau, cofilin-actin rods, and Hirano bodies which are related to the actin cytoskeleton. Cofilin is one of the most affluent and common actin-binding proteins and plays a role in cell motility, migration, shape, and metabolism. They also play an important role in severing actin filament, nucleating, depolymerizing, and bundling activities. In this review, we summarize the structure of cofilins and their functional and regulating roles, focusing on the synaptic dysfunction, β-amyloid plaques, hyperphosphorylated tau, cofilin-actin rods, and Hirano bodies of AD.

## Introduction

The cytoskeleton is the network structure of protein fibers in eukaryotic cells, including microfilaments, microtubules, and intermediate fibers (Mullins and Hansen, 2013). Among them, microfilaments (actin fibers) are composed of actin, which play an important role in many biological processes such as endocytosis (Akamatsu et al., 2020), extracellular secretion (Teitelbaum and Zou, 2011), cell migration (Yamaguchi and Condeelis, 2007), and axoplasmic movement (Kuznetsov et al., 2010). Therefoere, actin is one of the most abundant proteins in eukaryotic cells and is a major component of the cytoskeleton (Blanchoin et al., 2014). The assembly and disassembly of the actin cytoskeleton are essential for many cellular processes such as cell motility, migration, exocytosis and endocytosis, dendritic spine morphogenesis, intracellular transport, cell shape, and polarity (Pollard and Borisy, 2003; Porat-Shliom et al., 2013; Inagaki and Katsuno, 2017; Konietzny et al., 2017; Senju and Lappalainen, 2019). Actin has two different forms, the monomeric (G-actin) and the filamentous form (F-actin; Ampe and Van Troys, 2017). The G-actin polymerizes to form the filaments (F-actin) in various shapes. F-actin filaments are polar double-stranded helices with two ends, the barbed end and the pointed end. The polymerization of G-actin to F-actin is a dynamic process, regulated by several different pathways including the various actin-binding proteins (ABPs; Bamburg and Wiggan, 2003; Pollard and Borisy, 2003).

Actin-binding proteins interact with actin filaments and help with the formation, function, and restructuring of the actin cytoskeleton (Weaver, 2008; Albiges-Rizo et al., 2009; Gau et al., 2015; Madsen et al., 2015). Cofilins are an important ABP and consist of five members, including cofilin-1, cofilin-2, destrin, depactinm, and actophorin, that have been characterized in various organisms ranging from eukaryon to mammal (Ono et al., 1994; Yamashiro et al., 2005; Augustine et al., 2008; Ueno et al., 2010; Makioka et al., 2011). Cofilin-1, encoded by the *CFL1* gene, is widely distributed in various tissues and also known as the non-muscle isoform (Goodson et al., 2012). Cofilin-1 is one of the major regulators of actin dynamics due to its F-actin severing, depolymerizing, nucleating, and bundling activities (Bamburg and Bernstein, 2016). Cofilin-1 is also implicated in the proliferation, invasion, and metastasis of malignant cells (Lee et al., 2005; Wang et al., 2006, 2007; Tahtamouni et al., 2013; Lu et al., 2015; Tsai et al., 2015).

Since cofilin is ubiquitously expressed in the brain and excitatory synapses (Racz and Weinberg, 2006; Bellenchi et al., 2007; Görlich et al., 2011), increasing evidence suggests its critical role in spine morphology (Hotulainen et al., 2009; Gu et al., 2010; Rust et al., 2010; Bosch et al., 2014), synaptic plasticity (Zhou et al., 2004; Gu et al., 2010; Rust et al., 2010; Bosch et al., 2014), and neurotransmitter release as well as in learning and behavioral abnormalities (Rust et al., 2010; Goodson et al., 2012; Zimmermann et al., 2015). Due to the important role of synaptic function in brain function (Malenka, 1994; Zucker and Regehr, 2002; Südhof, 2013), any dysregulation or loss of synaptic function may lead to neurodegenerative diseases such as the Alzheimer’s disease (AD; Foster et al., 2017), Parkinson’s disease (Soukup et al., 2018), Huntington disease (Smith-Dijak et al., 2019), and autism (Perche et al., 2010). Neurodegenerative diseases severely impact patients’ quality of life and are currently a major public health concern. Therefore, understanding the role of cofilin can not only help elucidate the disease mechanism but also provide important insights for potential therapy.

## Structure, Function, and Regulation of Cofilin

Around 150 amino acid residues in the polypeptide chains of ADF/cofilins are known as the actin-depolymerizing factor homology domains (ADF-H domain), which consist of six-stranded mixed β-sheets, forming the specific three-dimensional structures (Lappalainen et al., 1998; Paavilainen et al., 2008). Cofilin-1 has a molecular weight of 19 kDa and contains an ADF-H domain which has several additional amino acid residues at the N-terminus of the polypeptide chain and about 10 amino acid residues at the C-terminus (Hild et al., 2014). ADF/cofilin from vertebrates also contains nuclear localization sequences (Shishkin et al., 2016). Cofilins is known to bind to actin, phosphatidylinositol 4, 5-bisphosphate (PIP2; Yonezawa et al., 1991), cortactin (Ivanov et al., 2004), and serine/threonine-protein kinase LIMK1 (Ivanovska et al., 2013). Low concentration of cofilins can sever the actin filaments and promote depolymerization whereas a high concentration of cofilins promotes actin nucleation and polymerization. Cofilin, directly or indirectly, nucleates the actin polymerization in a concentration-dependent manner (Bravo-Cordero et al., 2013). It can facilitate F-actin assembly via stabilizing preexisting filaments and nucleating new at high concentrations (Wang et al., 2007) whereas it can promote F-actin disassembly by accelerating the dissociation of monomeric actin from the filaments’ minus ends and severing the F-actin at lower concentrations (Albiges-Rizo et al., 2009; Madsen et al., 2015). Filament severing can lead to either a net assembly or disassembly of F-actin depending on the activity of actin polymerizing proteins and the local G-actin concentration (Gau et al., 2015). As a seven-subunit complex, the actin-related proteins 2/3 (ARP2/3) complex is involved in cofilin-regulating actin nucleation and filament branching of the ARP2/3 complex, which can bind to actin to supply nucleation and formation of the actin branches (dos Remedios et al., 2003; Winder and Ayscough, 2005). The ARP2/3 complex and cofilin synergistically generate the free barbed ends for the actin polymerization (Tania et al., 2013) when cofilin decreases the affinity of the ARP2/3 complex to the filaments and accelerates the dissociation of the old actin branches (Chan et al., 2009). The ARP2/3 complex and cofilin also play a role in the regulation of axonal growth cones (Dumpich et al., 2015). Cofilin is necessary for the dynamic changes in the cytoskeleton require for the axon re-engagement and myelination of Schwann cells (Sparrow et al., 2012). Other cellular functions of cofilin such as the regulation of nuclear integrity, nuclear actin monomer transfer, apoptosis, and lipid metabolism have been discussed in a review by Kanellos and Frame (2016).

The phosphorylation of a single cofilin residue Ser-3 prevents its binding to the F- and G-actin (Agnew et al., 1995; Moriyama et al., 1996), and only a dephosphorylated cofilin can elicit a biological response including actin-binding and nucleary. Therefore, their activity is regulated by phosphorylation/dephosphorylation. Interestingly, cofilin can also be phosphorylated at T25 regulated by TGF-β signaling (Zhu et al., 2009). And Y68 is identified as the major phosphorylation site of the tyrosine phosphorylation of cofilin by v-Src (Yoo et al., 2010). Also, proteomic studies identified new phosphorylation sites of cofilin e.g., Y82, T63, and S108. Generally, phosphorylation/dephosphorylation of cofilins is subject to signaling pathways involved in kinases and phosphatases, in response to the extracellular signals and changes in the microenvironment (Wang et al., 2007; Tomasella et al., 2014; Chang et al., 2015). The phosphorylation of cofilins is activated by LIM-kinases (LIMK1 and LIMK2) and testicular protein kinases (TESK1 and TESK2), whereas the dephosphorylation of cofilins is activated by the slingshot protein phosphatases (SSH1, SSH2, and SSH3), chronophin, and protein phosphatases 1 and 2A (PP1 and PP2A; Aragona et al., 2013). LIMK1 catalyzes the phosphorylation of a single cofilin residue Ser-3 (Morgan et al., 1993; Agnew et al., 1995; Moriyama et al., 1996), which inhibits the cofilin and actin-binding (Yang et al., 1998; Toshima et al., 2001). LIMK1 is regulated by the Rho kinase (ROCK) or the p21-activated protein kinase (Pak) signaling (Edwards et al., 1999). The phosphorylated cofilin binds to the scaffolding protein 14-3-3 and restricts the accessibility of the phospho-cofilin to the more general phosphatases (Gohla and Bokoch, 2002), however, not the slingshot phosphatases (Niwa et al., 2002; Huang et al., 2006; Eiseler et al., 2009). The slingshot phosphatase has three mammalian isoforms which dephosphorylate cofilin (Niwa et al., 2002). SSH-1L dephosphorylates cofilin requires SSH-1L to bind to F-actin, which can be prevented by the protein kinase D phosphorylation of SSH-1L at Ser-978 (Eiseler et al., 2009; Peterburs et al., 2009). Moreover, the phosphorylation of SSH-1L is isolated by isoforms of 14-3-3, which inhibits the translocation to the sites for its activation (Nagata-Ohashi et al., 2004). Interestingly, the dephosphorylation of SSH-1L by calcineurin (Wang et al., 2005) also requires F-actin binding, which promotes the cofilin activation to cellular sites where the F-actin levels are relatively high (Nagata-Ohashi et al., 2004; Soosairajah et al., 2005; Kurita et al., 2008). Specifically, SSH-1L dephosphorylates the LIMK in an activation loop in order to inhibit its phosphorylation of cofilin (Soosairajah et al., 2005).

In addition, the activity of cofilins is also regulated by pH *in vitro* (Yonezawa et al., 1985) and *in vivo* (Bernstein et al., 2000). However, pH sensitivity is not universally applicable for all of the ADF/cofilins in different species. For example, cofilin-1 and cofilin-2 activity are pH-independent only in the mice (Vartiainen et al., 2002). Interestingly, the activity of cofilins can be regulated by the direct binding of phosphatidylinositols, especially the PIP2, which prevents binding to actin (Yonezawa et al., 1991). Therefore, the balance between the membrane-bound and the free active ADF/cofilins can be modulated by the alterations in the PIP2 density of the cellular membrane (Zhao et al., 2010). Other proteins also can directly or indirectly modulate the cofilin activity through the interaction of ADF/cofilins for example coronin promotes actin filament severing by the recruitment of cofilin to the filament side (Mikati et al., 2015). Cofilin is inactivated by cortactin binding which is important for actin-based dynamic protrusions, invadopodia, and podosomes (Beaty and Condeelis, 2014; Zalli et al., 2016). The cyclase-associated protein 1 (CAP1) and actin-interacting protein 1 (AIP1) facilitate the disassembling of the cofilin-bound actin filaments (Zhou et al., 2014; Nomura et al., 2016). The cellular redox state also modulates the activity of ADF/cofilins. The cofilin activity is influenced by redox-related modifications of Cys residues through the disulfide bonds (Klemke et al., 2008), *S*-glutathionylation (Fratelli et al., 2002), and *S*-nitrosylation (Zhang et al., 2015). Oxidized cofilin is dephosphorylated at Ser-3 which in turn impairs the actin depolymerizing function (Klemke et al., 2008). Therefore, cofilins acts on a multifaceted role in cells implicated in different pathological processes.

## Role of Cofilin in AD

Alzheimer’s disease characterized by cognitive impairment, including memories, and is the most frequent cause of dementia affecting about 24 million people worldwide (Foster et al., 2017; Soukup et al., 2018; Smith-Dijak et al., 2019). Before the emergence of cognitive impairment, symptoms such as thinning of the cortex, accumulation of β-amyloid, and decreased hippocampal volume are common. Hence, the accumulation of β-amyloid and hyperphosphorylated tau are two pathological hallmarks in AD brains (Goedert, 2010; Holtzman et al., 2012). Furthermore, another proteinopathies are also invovled in brains of AD containing α-synuclein-containing Lewy bodies (Kosaka et al., 1984), cofilin-actin rods (Bamburg and Bloom, 2010), Hirano bodies (Galloway et al., 1987; Maciver and Harrington, 1995), and TDP-43 inclusions (Josephs et al., 2015). More importantly, the cellular biological signatures of AD, including the synaptic dysfunction (Jack and Holtzman, 2013), β-amyloid plaques (Holtzman et al., 2012), hyperphosphorylated tau (Goedert, 2006), cofilin-actin rods (Bamburg and Bloom, 2009), and Hirano bodies (Galloway et al., 1987; Maciver and Harrington, 1995) have been identified. Here we discuss the role of cofilin in such biological processes.

### Role of Cofilin in Synaptic Dysfunction

Decreased glucose utilization has been observed in humans through PET imaging before the emergence of overt symptoms, suggesting that synaptic dysfunction precedes the AD pathogenesis (Jack and Holtzman, 2013; Jack et al., 2013). Synaptic function is multifaceted and relies on factors such as spine morphology, synaptic plasticity, neurotransmitter release, learning, and so on (Richmond, 2005).

A previous study demonstrated that AD is related to changes in spine morphology (Blanpied and Ehlers, 2004). Loss of dendritic spines has been observed in primary neurons from AD mice (Herms and Dorostkar, 2016), mouse models of AD (Spires-Jones et al., 2007; Wu et al., 2010; Herms and Dorostkar, 2016), and postmortem brain tissues from AD patients (Tsai et al., 2004; Spires et al., 2005; Spires-Jones et al., 2007). Loss of dendritic spines leads to the impairment of synaptic transmission as the dendritic spines are the primary sites for receiving information and cellular substrates for synaptic plasticity (Cummings et al., 2015). F-actin is the main cytoskeletal protein found in the dendritic spines (Matus et al., 1982; Cohen et al., 1985) and its depolymerization is dynamically modulated by cofilin-1 (Okamoto et al., 2007; Hotulainen et al., 2009; Bamburg and Bernstein, 2010, 2016; Bernstein and Bamburg, 2010; Gu et al., 2010), which is vital for regulating the spine formation and elimination, and the synaptic-activity-dependent structural changes (Star et al., 2002; Okamoto et al., 2007; Korobova and Svitkina, 2010). And the polymerization of actin is inducedby Arp2/3, mDia2, WAVE complex, etc. (Welch et al., 1997; Beli et al., 2008). The actin filament disassembly induced by ADF/cofilin maintains the proper spine length and morphology (Hotulainen et al., 2009). In the initial phase of remodeled spine substructures, active cofilin is delivered to the spine and cofilin subsequently forms a stable complex with F-actin in order to remain at the spine and consolidate the spinal expansion during the stabilization phase (Bosch et al., 2014). Meanwhile, cofilin-1 is inactivated through the increase in phosphorylation at Ser3 accompanied by alteration of dendritic spine morphology in the dentate gyrus (Nader et al., 2019). Active cofilin, induced by the role of dephosphorylation, binds to actin, and facilitates the conversion of the F-actin to G-actin (Bamburg and Bernstein, 2016). The Rac1/cofilin pathway is inactivated through suppression of the cofilin phosphorylation which causes the loss of dendritic spine in the hippocampus (Yang et al., 2018). The density of dendritic spines and dendritic complexity increase, similar to the change of phosphorylation and redistribution of cofilin-1 in an AD rat model (Han et al., 2017). Cofilin-1 knockdown leads to a decrease number of thin spines and decrease length of dendritic protrusions (Hotulainen et al., 2009). Furthermore, both the spinal head width and length are augmented in primary hippocampal cultures, as observed in the cofilin-1 mutant mice (Rust et al., 2010). An increased spinal density and enlargement in the hippocampal slices from cofilin-1 mutant mice have also been observed (Rust et al., 2010). Mature spines and spinal densities are enhanced when the constitutively inactive cofilin-1 is overexpressed in the hippocampal cultures (Shankar et al., 2007; Gu et al., 2010).

Spines possess plasticity undergoing long- and short-term modifications of the input activity (Nimchinsky et al., 2002). Long-term potentiation (LTP) and long-term depression (LTD) are usually linked with learning and memory functions (Malenka and Bear, 2004). Both pre- and post-synaptic modulations take place. The strength of the presynaptic transmission is modified by alteration in neurotransmitter release, whereas the change in type, number, and property of receptors are implicated in the modification of postsynaptic strength (Malenka, 1994; Collingridge et al., 2004; Richmond, 2005). Cofilin-1 plays an important role in dendritic spine morphology and structural plasticity as their distributional areas covers the dynamic F-actin network, which is responsible for the spine morphological changes during synaptic plasticity (Racz and Weinberg, 2006; Honkura et al., 2008). Phosphorylation and dephosphorylation of ADF/cofilin are also associated with the formation of LTP, increased spine head volume (Fukazawa et al., 2003; Chen et al., 2007; Fedulov et al., 2007; Bosch et al., 2014), and the LTD and spine shrinkage (Zhou et al., 2004). Increased phosphorylated ADF/cofilin is related to the spine enlargement when LTP or learning (Chen et al., 2007; Fedulov et al., 2007). The immature spine features, induced by the cofilin-1 overexpression, resembles the N-methyl-D-aspartic acid receptor (NMDAR)-mediated LTD evoked by the low-frequency stimulations (Shi et al., 2009; Pontrello et al., 2012). The phosphorylation levels of ADF/cofilin increase when LTP is induced by the NMDAR stimulations that enhance the synaptic F-actin content, and stabilize and enlarge the dendritic spines (Fukazawa et al., 2003; Chen et al., 2007).

Docking and priming are the two vital presynaptic steps that ensure that the Ca^2+^-triggered synaptic vesicle (SV) exocytosis occurs successfully (Sudhof, 2004; Imig et al., 2014). Docking and priming are enhanced in the double mutants lacking ADF and cofilin-1, which increased the synaptic vesicle exocytosis (Wolf et al., 2015; Zimmermann et al., 2015). Interestingly, cofilin-1 also plays a vital role in a-amino-3-hydroxy-5-methyl-4-isoxazole propionic acid receptors’ (AMPARs) trafficking and accumulation (Gu et al., 2010; Rust et al., 2010; Wang et al., 2013). Cofilin-1 regulates the synaptic strength through AMPARs mobility through an actin-dependent mechanism but not its function in structural plasticity (Rust et al., 2010). However, cofilin-1 acts on the AMPARs accumulation during synaptic plasticity where LTP is found reduced in the cofilin-1 mutant mice (Rust et al., 2010), similar to the study that demonstrated that ADF/cofilin activity can influence the synaptic accumulation of glutamate receptor 1 (GluR1) and 2 (GluR2) in the rat infralimbic cortex (Wang et al., 2013). Moreover, the inactivation of cofilin-1 controls the AMPARs subunit GluR1’s synaptic accumulation during the chemical LTP (Gu et al., 2010). Overall, the cofilin-1 activity during LTP is related to the synaptic AMPARs accumulation.

As spine morphology, synaptic plasticity, and neurotransmitter release are heavily implicated in learning and memory, cofilin-1 also plays an important role in the learning and/or memory processes. Associative learning is found impaired in principal neurons of the adult telencephalon in cofilin-1 mutant mice (Rust et al., 2010). The levels of phosphorylated ADF/cofilin are also elevated in the rat hippocampal CA1 region during unsupervised learning in an enriched environment, suggesting that ADF/cofilin is vital for spatial learning (Fedulov et al., 2007). Furthermore, long-term spatial memory is reduced in cofilin-1 mutants whereas short-term spatial working memory is not fully impaired (Rust et al., 2010; Zimmermann et al., 2015). This finding is confirmed through the two distinct mechanisms for spatial memory (Bannerman and Sprengel, 2010). Furthermore, increased ADF/cofilin activity expedites the conditioned taste aversive memory through the regulation of synaptic AMPARs concentrations (Wang et al., 2013). More importantly, ADF/cofilin-mediated AMPARs trafficking also controls both the memory acquisition and extinction (Rust et al., 2010; Wang et al., 2013).

### Role of Cofilin in Aβ

Aβ is produced from the amyloid precursor protein (APP) and is one of the most primary hallmarks of AD. APP can be cleaved through two competing pathways, the non-amyloidogenic and the amyloidogenic pathways (Verdile et al., 2004). In the amyloidogenic pathways, APP is cleaved by the β-site APP cleaving enzyme 1 (BACE1) and γ-secretase, which produces the Aβ peptide (Aβ_1__–__40_ or Aβ_1__–__42_). In the non-amyloidogenic pathways, APP is cleaved by α-secretase to produce the non-amyloidogenic fragments (soluble APPα; sAPPα). The amyloid cascade hypothesis is the basic theory behind the pathological accumulation of AD (Hardy and Higgins, 1992). Aβ peptides assemble to form the extracellular amyloid plaques which cause neuronal death and a decline of cognitive functions. The amyloid plaques are observed in both the postmortem AD brain tissues and live AD human brains (Thompson et al., 2007; Ikonomovic et al., 2008).

Aβ plays a crucial role in cofilin deregulation through the LIMK1 pathways. Heredia et al. demonstrated that Aβ_1__–__40_ and Aβ_25__–__35_ fibrils induce the activation of LIMK, which leads to cofilin inactivation (Heredia et al., 2006). However, another study demonstrated, through the injection of Aβ_1__–__40_ fibrils into rat brains, that cofilin increased activation rather than inactivation (Bie et al., 2018). Furthermore, Ariadna et al. showed that LIMK1 was activated by Aβ_1__–__42_, which is paradoxically related to the increased cofilin activation. Therefore, there may exist other pathways that are involved in cofilin deregulation via Aβ such as the SSH1 pathway (Kim et al., 2009; Mendoza-Naranjo et al., 2012). The cofilin activation is facilitated by the RanBP9 through the SSH1 pathway with increasing levels of SSH1 in the primary neurons and the brain (Woo et al., 2015a). RanBP9 is a scaffolding protein that promotes the production of Aβ (Lakshmana et al., 2009). It is highly expressed in APP transgenic mice (Woo et al., 2012, 2015a) and the brains of human AD patients (Lakshmana et al., 2010). Furthermore, the level of cofilin activation is enhanced in AD brains while accompanied with no alterations of the phosphoLIMK1 levels (Kim et al., 2013). Moreover, cofilin-actin pathology is alleviated by the genetic deletion of RanBP9 in the APP/PS1 transgenic mice (Woo et al., 2015a). Kim et al. found that Aβ oligomer receptor LilrB2 mediates cofilin activation through the Aβ_1__–__42_ oligomers (Kim et al., 2013), however, Woo et al. (2015b) showed that β1-integrin conformers mediate the activation of cofilin induced by the Aβ_1__–__42_ oligomers through the SSH1 pathway. In addition, Aβ selectively impairs the metabotropic glutamate receptor 7 (mGluR7) regulation of NMDARs by decreasing the cofilin-mediated actin depolymerization through a p75^NTR^-dependent mechanism and increasing the Pak activity (Gu et al., 2014). The phospho-cofilin decreases during the Aβ early pathology progression while increasing during the mid-late pathology (Barone et al., 2014).

### Role of Cofilin in Tau

Tau is a microtubule-associated protein that plays an important role in microtubule polymerization and axonal transport (Buée et al., 2000). The hyperphosphorylated or abnormally phosphorylated tau aggregates intracellularly and is another major hallmark of AD (Benzing et al., 1993). The hyperphosphorylated tau forming the intracellular neurofibrillary tangles is first observed in the hippocampus and entorhinal cortex, and then in the neocortical regions (Braak and Braak, 1995).

Cofilin may precipitate together with tau in cofilin-actin rods as the 12E8 antibody that reacts with a class of cofilin-positive rods, which recognizes the pSer262/pSer356 of tau (Whiteman et al., 2009). Both the cofilin-actin aggregations and phospho-tau containing neuropil threads are highly expressed in AD brains, although they do not colocalize in the same brains of AD patients (Rahman et al., 2014). In APP/PS1 transgenic mice, cofilin-microtubule complexes increase and are accompanied by decreasing tau-microtubule complexes, which can be inhibited by *cofilin* genetic deletion (Woo et al., 2019). Cofilin also plays an important role in replacing tau from tubulin/microtubules, preventing tau-induced microtubule assembly both *in vitro* and *in vivo* (Woo et al., 2019). The tau hyperphosphorylation is relieved by the genetic reduction of *cofilin* in TauP301S (PS19) mice. Therefore, cofilin activated by dephosphorylation replaced the tau from microtubules that results in the tau hyperphosphorylation and inhibition of the tau-mediated microtubule dynamics.

### Cofilin-Actin Rods

Enrichment of cofilin-actin is also a pathological feature of AD (Maloney and Bamburg, 2007). Cofilin-actin can form the rod-shaped bundles of filaments, known as the cofilin-actin rods, that include ADF/cofilin:actin in a 1:1 molar ratio in cultured primary neurons or cell lines (Minamide et al., 2010). Moreover, the phospho-cofilin antibody cannot be immunostained within the rods, which indicates that the active cofilin is only present in the cofilin-actin rods (Maloney et al., 2005). Neuronal stress, such as ATP-depletion, peroxide, and glutamate rapidly induce the production of cofilin-actin rods (Minamide et al., 2000; Davis et al., 2009). The cofilin-actin rods formed by the neuronal stress are tandemly arranged where both concentrations of cofilin and actin are high (Minamide et al., 2000). The tandem arrays of cofilin are observed in the frontal cortex and hippocampus of human AD (Minamide et al., 2000) as well as in the transgenic AD mice models (Maloney et al., 2005). Moreover, cofilin-actin rods can also form in the organotypic hippocampal slices (Davis et al., 2009) and dissociated cultures containing dendrites and axons of the mice and rat cortical and hippocampal neurons (Minamide et al., 2000). Cofilin-actin rods have also been observed in the human AD brain using an electron microscope (Sisodia and Price, 1995).

Since the majority of neuronal stress agents induce a decline in ATP, it is the major neuronal stress that caused the formation of cofilin-actin rods. Rods are induced through the external application of ATP in some neurons (Homma et al., 2008). The disruption of mitochondrial electron transport leads to ATP-depletion and chronophin release from inhibition through its intracellular complex with Hsp90 (Huang et al., 2008). Chronophin is one of the halogen acid dehalogenase phosphatases that dephosphorylate the cofilin. Apart from chronophin, slingshot is another important de-phosphorylase. After its release from the inhibitory binding partner 14-3-3, the slingshot is dephosphorylated through an increasing calcium binding to the calmodulin; the complex stimulates calcineurin (Wang et al., 2005; Kim et al., 2009). The 14-3-3 is a family of regulatory proteins, highly expressed in the brain, and is required for hippocampal LTP and associative learning and memory functions (Qiao et al., 2014). Inhibition of 14-3-3 proteins leads to schizophrenia-related behavioral phenotypes and synaptic defects in mice (Foote et al., 2015). Furthermore, slingshot phosphatase may act as a subordinate role as the genetic knockdown of chronophin can retard the cofilin dephosphorylation and rod formation (Huang et al., 2008). In addition, the inhibitory binding partner 14-3-3 also plays a vital role in peroxide to induce the formation of rods via sulfhydryl oxidation of the 14-3-3 and its release of the active slingshot (Kim et al., 2009). The active cofilin is increased through dephosphorylation accompanied by the production of reactive oxygen species (ROS), which are essential for cofilin oxidation and rod formation (Minamide et al., 2000; Bernstein et al., 2012). Meanwhile, increased ROS favors cofilin saturation region of F-actin, which is readily reduced and producing small stable fragments (Chen et al., 2015). The small stable fragments may promote the formation of rods, which may directly bind these fragments and intermolecular disulfide cross-linking of cofilin or may be cofilin or cofilin-actin formed through the oxidative dimerization of actin and dimer involved in the filament assembly and binding (Minamide et al., 2010; Bernstein et al., 2012).

Around 20% of the total population is formed as rods in the cultured rat hippocampal neurons treated with Aβ_1__–__42_ (Maloney et al., 2005). However, rod formation is slower in human cortical neurons treated with synthetic Aβ_1__–__42_ oligomers (sAβ_1__–__42_; Deshpande et al., 2006). The rods only form within the soma and neurites of the neurons that have Aβ treatment-induced activated cofilin (Maloney et al., 2005). There exist some persistent rods that form within the 24 h of a transient 30 min ATP-depletion and washout, whereas other rods induced by Aβ, are reversible and disappear completely by 24 h after the washout (Davis et al., 2009). The insufficient recovery of mitochondrial function in impaired neurites may lead to the difference in the rod persistence (Minamide et al., 2000). In addition, microinjection of cofilin results in rod formation in neurons *Aplysia kurodai*, assessed using electrophysiological methods (Jang et al., 2005).

### Hirano Bodies

Cofilin-actin enriches as cytoplasmic para-crystalline lattices that form the Hirano bodies. Hirano bodies are an ordered array of parallel regularly spaced 6–10 nm filaments in the orthogonal layers encircled by a region of less structured dense actin (Schochet and McCormick, 1972; Tomonaga, 1974). Hirano bodies mainly contain ADF, cofilin, and actin (Maciver and Harrington, 1995). Hirano bodies are significantly increased in AD patients compared to aged-matched individuals (Schmidt et al., 1989). Hirano bodies are frequently distributed in the Sommer’s sector of Ammon’s horn (Hirano, 1994), although they are found in several areas of the brain. Sommer’s sector of Ammon’s horn is enriched with Pick bodies and AD neurofibrillary tangles, which are related to the development of new memories. There, the formation of Hirano bodies in this region impairs cognition in neurodegenerative disorders including AD (Hirano, 1994). Phalloidin that recognizes F-actin, but not the cofilin, stains the Hirano bodies (Galloway et al., 1987). Hirano bodies comprise of epitopes of tau, ADF/cofilin, and actin-associated proteins (Galloway et al., 1987; Peterson et al., 1988; Maciver and Harrington, 1995), however, the mechanism of the formation of Hirano bodies is still unclear. Fortunately, an identical morphology between Hirano bodies and the structure is expressed in the mammalian cells of a C-terminal (CT) fragment of actin cross-linking protein in the *Dictyostelium discoideum* (Maselli et al., 2002). Therefore, a potential model system exists for further study (Davis et al., 2008).

## Conclusion

**FIGURE 1:**
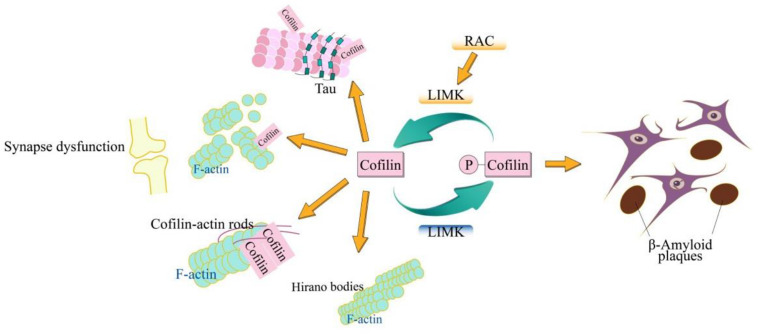
Schematic model of cofilin in AD. The cofilin is phosphorylated by LIMK and dephosphorylated via SSH. The dephosphorylated (active) cofilin is closely related to the cellular biological signatures of AD, which include synaptic dysfunction, β-amyloid plaques, hyperphosphorylated tau, cofilin-actin rods, and Hirano bodies. The cofilin plays an important role in synaptic function through its action in spine morphology, synaptic plasticity, neurotransmitter release, and learning. Cofilin-actin can form the rod-shaped bundles of filaments (cofilin-actin rods) and cytoplasmic para-crystalline lattices (Hirano bodies).

Cofilin plays a vital role in ctin filament dynamics and reorganization through severing actin filaments, which is regulated by several mechanisms including ARP2/3 complex, phosphorylation on Ser3, pH, phosphatidylinositols and so on. As a common neurodegenerative disorder, AD has complicated various cellular biological processes. The typical molecular signatures of AD include the synaptic dysfunction (Jack and Holtzman, 2013), β-amyloid plaques (Holtzman et al., 2012), hyperphosphorylated tau (Goedert, 2006), cofilin-actin rods (Bamburg and Bloom, 2009), and Hirano bodies (Galloway et al., 1987; Maciver and Harrington, 1995; Figure 1). Cofilin can regulate the depolymerization of F-actin dynamically, which is important for regulating the spine formation and elimination, and the synaptic-activity-dependent structural changes, thus acting the synaptic dysfunction. Aβ plays a crucial role in cofilin deregulation through the LIMK1 pathways. Cofilin activated by dephosphorylation replaced the tau from microtubules that results in the tau hyperphosphorylation and inhibition of the tau-mediated microtubule dynamics. Cofilin-actin rods and hirano bodies, being associated with the actin cytoskeleton are also associated with AD. More importantly, all these molecular signatures of AD are not unique but are related to one other. In primary neurons, cofilin and tau are necessary for the Aβ-induced synaptic and mitochondrial dysfunction. Moreover, either reduction of *cofilin* or *tau* rescues the synaptic plasticity and memory defects in APP transgenic mice (Rapoport et al., 2002; Roberson et al., 2007; Jin et al., 2011; Shipton et al., 2011; Woo et al., 2015b). Cofilin and calcineurin modulate the Aβ-induced dendritic spine loss, which demonstrates that SSH1 is implicated in Aβ-induced cofilin activation (Shankar et al., 2007). The dephosphorylation of SSH1, mediated by calcineurin, activates cofilin which allows SSH1 to avoid inhibition via the 14-3-3 proteins (Wang et al., 2005; Eiseler et al., 2009; Kim et al., 2009). The knockdown of SSH alleviates the neurotoxic effects of Aβ_1__–__42_ oligomers and mitochondrial translocation of activated cofilin (Woo et al., 2015b). Furthermore, the formation of the cofilin-actin rod, requiring cofilin activation, is induced by bioactive Aβ dimers/trimers treatment in neurites of only ∼20–30% of the cultured hippocampal primary neurons, whereas the formation of the cofilin-actin rod is enhanced in the mossy fiber track and the dentate gyrus, but not the CA regions (Davis et al., 2011). Furthermore, direct cofilin oxidation is necessary for cofilin-actin rod assembly (Bernstein et al., 2012), and mitochondrial translocation of cofilin (Klamt et al., 2009). F-actin-containing rod-like structures include the hyperphosphorylated tau in FTDP-17 transgenic mice and *Drosophila* (Fulga et al., 2007). Thus, cofilin as an early marker in the AD, is an ideal target for therapeutic intervention that might be useful in treatment of AD even in many different neurological diseases. Therefore, a systematic understanding of the role of cofilins in these molecular characteristics not only is helpful for the understanding of AD and but also lays a molecular foundation for the treatment of AD.

## Author Contributions

QW, WY, XY, YW, and YL collected the data. QW wrote the article. HQ reviewed the article. All authors contributed to the article and approved the submitted version.

## Conflict of Interest

The authors declare that the research was conducted in the absence of any commercial or financial relationships that could be construed as a potential conflict of interest.
